# Editorial: Community series in translational insights into mechanisms and therapy of organ dysfunction in sepsis and trauma - volume III

**DOI:** 10.3389/fimmu.2024.1447892

**Published:** 2024-07-11

**Authors:** Borna Relja, Pietro Ghezzi, Sina M. Coldewey, Valentin A. Pavlov, Madhav Bhatia, Bettina Jungwirth, Christoph Thiemermann

**Affiliations:** ^1^ Department of Trauma, Hand, Plastic and Reconstructive Surgery, Translational and Experimental Trauma Research, University Hospital Ulm, Ulm University, Ulm, Germany; ^2^ University of Urbino Carlo Bo, Urbino, Italy; ^3^ University Hospital Jena, Jena, Germany; ^4^ Feinstein Institutes for Medical Research, Manhasset, NY, United States; ^5^ Department of Pathology and Biomedical Science, University of Otago, Christchurch, New Zealand; ^6^ Department of Anesthesiology and Intensive Care Medicine, University Hospital Ulm, Ulm University, Ulm, Germany; ^7^ Queen Mary University of London, London, United Kingdom

**Keywords:** critical care (7/8), organ failure, therapy, diagnosis, mechanisms, machine learning

Translational research on trauma, sepsis, and multiple organ damage is continuously evolving, fostering advancements in the development of diagnostics and therapeutics, and improving our understanding of the complex pathophysiological mechanisms underlying these life-threatening conditions. The contributors of this Research Topic explore the different aspects of the pathophysiology of trauma and critical illness. With different approaches, these studies describe novel potential diagnostic markers, innovative therapeutic interventions, and elucidate the complex mechanisms underlying immune responses and organ damage in patients with trauma, sepsis and/or multiple organ failure.

Life-threatening organ dysfunction and failure in trauma and critically ill patients stem from a dysregulated host response to infection and inflammation. Despite advancements in understanding the key signaling pathways involved, translating this knowledge into organ-protective therapies remains challenging. Current treatments primarily rely on infection control, antibiotics, supportive care, and early goal-directed therapy.

Trauma and sepsis-induced organ dysfunction are driven by excessive systemic inflammation, secondary to tissue damage and ischemia-reperfusion injury. The endothelial glycocalyx is a critical early site involved in triggering a pro-inflammatory response. Alterations in its structure lead to the release of degradation products and damage-associated molecular patterns (DAMPs) that, along with pathogen-associated molecular patterns (PAMPs), amplify systemic inflammation and, hence, contribute to organ dysfunction including acute kidney injury, respiratory failure, and cardiomyopathy.

## Three major fields of research have been covered by the authors

First, in terms of sepsis diagnosis and treatment, Dennhardt et al. investigate the clinical significance of circulating cell-free DNA (cfDNA) levels in sepsis patients as predictors of both mortality and sepsis-associated acute kidney injury, suggesting elevated cfDNA levels as a potential enhancer of risk assessment in sepsis. Similarly, Qiang et al. explore the therapeutic potential of progesterone in sepsis, presenting promising results in septic mice and patients by inhibiting procathepsin-L-mediated inflammation. Guggemos et al. investigate the impact of specific polymorphisms of the ATP-sensitive P2X7 receptor on sepsis susceptibility and prognosis, revealing their potential role as genetic markers. Wakeley et al. examine the role of Herpes virus entry mediator in neonatal sepsis response, highlighting its importance in vascular and hemodynamic resilience, while Kasper et al. study the effects of hemadsorption therapy on endothelial barrier dysfunction in sepsis *in vitro*, indicating its potential transient clinical improvement.

Second, with a focus on trauma, injury, and organ damage, Horner et al. explore the immune suppressive role of DAMPs in sterile traumatic injury, proposing potential therapeutic strategies to mitigate immune suppression in their review. Wolfschmitt et al. investigate the impact of hyperoxemia on the metabolism of circulating immune cells during intensive care, focusing on granulocytes in models of acute subdural hematoma and hemorrhagic shock. Groven et al. explore the association between circulating miRNA expression in extracellular vesicles (EVs) as biomarkers for specific injuries following multiple trauma and surgical invasiveness. Meng et al. investigate how aging influences liver inflammation and damage after trauma, highlighting dysregulated immune responses, while Zhou et al. examine the exacerbation of lung damage after trauma in aged mice, focusing on the role of NF-κB and inflammasomes. Neu et al. explore the cardiovascular impairment in a murine model of hemolytic-uremic syndrome induced by Shiga toxin, a model which replicates the extrarenal manifestations observed in HUS patients. Fachet et al. propose a feature-based risk assessment system for predicting hyperinflammatory patterns and infectious outcomes in polytrauma patients using predictive machine learning modeling.

The third part of this Research Topic addresses therapeutic interventions and drug repurposing. Verra et al. investigate the effects of baricitinib, a JAK1/JAK2 inhibitor, on sepsis-induced cardiac dysfunction and multiple-organ failure, suggesting its potential for trauma-associated sepsis. Hof et al. study the effects of carbachol on gastric and oral microcirculation during hemorrhagic shock in dogs, highlighting its potential therapeutic use in improving tissue oxygenation. Yamaga et al. discuss potential therapeutic strategies targeting DAMPs to alleviate radiation-induced injury, focusing on the mechanisms of DAMP release and their detrimental effects on the immune system. To advance our understanding of critical illness, Li et al. identify key genes and pathways associated with DNA damage responses, inflammation, and cellular senescence in elderly patients with acute respiratory distress syndrome, offering insights into potential diagnostic biomarkers and therapeutic targets. Murao et al. utilize single-cell RNA sequencing to investigate immune cell responses in sepsis, providing insights into distinct immune cell subsets and their roles in inflammation and tissue repair, thus enhancing our understanding of the pathophysiology of sepsis. Wang et al. conducted the LiBOD study to assess the potential of serum-derived EVs as biomarkers for decision-making in polytrauma cases, suggesting specific EVs as valuable biomarkers for assessing and monitoring the severity of polytrauma and associated organ damage.

These studies collectively contribute to a better understanding of diagnostic markers, therapeutic interventions, immune responses, and the mechanisms underlying organ injury and dysfunction in trauma, sepsis, and related conditions, offering potential avenues for improving care and outcomes of patients with critical illness.

As we review the research presented in this Research Topic, it is clear how scientific research significantly shapes the field of trauma and critical care medicine, addressing the issue of improving patient outcomes, moving towards precision medicine in intensive care.

We conclude this Research Topic with deep gratitude to the researchers, clinicians, and patients whose dedication has made this work possible. We hope the insights from these studies will be interesting and educational to our readers and help developing this area of research on a challenging condition.

## Author contributions

BR: Writing – original draft. PG: Writing – review & editing. SC: Writing – review & editing. VP: Writing – review & editing. MB: Writing – review & editing. BJ: Writing – review & editing. CT: Writing – review & editing.

